# Suppression of HIV Replication by CD8^+^ Regulatory T-Cells in Elite Controllers

**DOI:** 10.3389/fimmu.2016.00134

**Published:** 2016-04-18

**Authors:** Wei Lu, Song Chen, Chunhui Lai, Mingyue Lai, Hua Fang, Hong Dao, Jun Kang, Jianhua Fan, Weizhong Guo, Linchun Fu, Jean-Marie Andrieu

**Affiliations:** ^1^Institut de Recherche sur les Vaccins et l’Immunothérapie des Cancers et du Sida, Université de Paris Descartes, Paris, France; ^2^Sino-French Collaborative Laboratory, Tropical Medicine Institute, Guangzhou University of Chinese Medicine, Guangzhou, China; ^3^Xishuangbanna Center for Disease Control and Prevention, Jinghong, China

**Keywords:** AIDS, elite controllers, CD4^+^ T-cells, suppressive/regulatory CD8^+^ T-cells, NK cells, HLA-B:Bw4-80Ile gene, KIR3DL1-expressing CD8^+^ T-cells, HIV-1 suppression assay

## Abstract

We previously demonstrated in the Chinese macaque model that an oral vaccine made of inactivated SIV and *Lactobacillus plantarum* induced CD8^+^ regulatory T-cells, which suppressed the activation of SIV^+^CD4^+^ T-cells, prevented SIV replication, and protected macaques from SIV challenges. Here, we sought whether a similar population of CD8^+^ T-regs would induce the suppression of HIV replication in elite controllers (ECs), a small population (3‰) of HIV-infected patients with undetectable HIV replication. For that purpose, we investigated the *in vitro* antiviral activity of fresh CD8^+^ T-cells on HIV-infected CD4^+^ T-cells taken from 10 ECs. The 10 ECs had a classical genomic profile: all of them carried the KIR3DL1 gene and 9 carried at least 1 allele of HLA-B:Bw4-80Ile (i.e., with an isoleucine residue at position 80). In the nine HLA-B:Bw4-80Ile-positive patients, we demonstrated a strong viral suppression by KIR3DL1-expressing CD8^+^ T-cells that required cell-to-cell contact to switch off the activation signals in infected CD4^+^ T-cells. KIR3DL1-expressing CD8^+^ T-cells withdrawal and KIR3DL1 neutralization by a specific anti-killer cell immunoglobulin-like receptor (KIR) antibody inhibited the suppression of viral replication. Our findings provide the first evidence for an instrumental role of KIR-expressing CD8^+^ regulatory T-cells in the natural control of HIV-1 infection.

## Introduction

The success of antiviral vaccines developed over the past decades generally depends on their ability to mimic a protective immunity similar to that induced by natural infection. In this context, an improved understanding of the host factors associated with the natural control of viral replication in elite controllers (ECs), a small group (3‰) of HIV-1 infected patients with long-term natural control of their viral replication (1), would potentially provide important information relevant for the development of new vaccines against HIV.

So far, the immunological features conferring protection against HIV in ECs have remained largely unknown. Specific HLA class I-B alleles (including HLA B27 and B57), gathered under the serological HLA-Bw4 motif, have been reported to be constantly associated with a favorable outcome of HIV-1 infection (2–4). This genetic association is in agreement with the immunogenetic understanding that epistatic interactions between killer cell immunoglobulin-like receptors (KIRs) and their cognate HLA-I ligands determine the outcome of viral infections and susceptibility to autoimmune diseases and cancer (5–7).

HLA-B molecules display one of two mutually exclusive serological epitopes, Bw4 and Bw6, which are identified on the basis of five variable amino acids at positions 77 and 80–83 (8). About two thirds of all HLA-B molecules express Bw6, whereas Bw4 is present in the remaining third and in several HLA-A molecules. The HLA-Bw4 motif, which is expressed by leukocytes, including CD4^+^ T-cells, the principal target of HIV, is a ligand of KIRs (5, 9). KIRs constitute a polygenic and polymorphic family of receptors, with both inhibiting and activating motifs (10) that are expressed by NK cells (11) and T cells (12–15), and participate in both innate and adaptive immunity. NK cells were reported to inhibit HIV-1 replication in CD4^+^ T-cells in a HLA-B:Bw4-80Ile (i.e., with an isoleucine (Ile) residue at position 80) and KIR3DS1- or KIR3DL1-dependent manner (16, 17). Inhibition of viral replication by NK cells, most likely involved in the initial viral control of primary HIV-1 infection, is however modest (18, 19). Surprisingly, little information is available on the role of KIR-expressing CD8^+^ T-cells in the containment of HIV-1 replication during the natural course of the infection. Here, we investigated the role of KIR-expressing CD8^+^ T-cells in the control of viral replication in CD4^+^ T-cells of 10 HIV-1-infected ECs and examined its association with either amino acid polymorphism of HLA alleles or KIR genotype. Ten untreated patients with high viral load (HVLpts) and two healthy donors (HDs) served as controls. We found that fresh KIR3DL1-expressing CD8^+^ T-cells from 9 out of the 10 examined ECs were capable of strongly suppressing HIV-1 replication in HIV-1-infected CD4^+^ T-cells carrying an HLA-B:Bw4-80Ile motif.

## Materials and Methods

### Patients

Ten ECs with an undetectable plasma viral load (<50 copies/ml) for ≥10 years and 10 HIV-1-infected HVLpts (>10^4^ copies/ml) (Table S1 in Supplementary Material) were recruited from a cohort of untreated individuals infected with HIV-1 CRF01_AE subtype at the CDC of Xishuangbanna (Yunnan, China) (20, 21). The study was approved by the institutional review boards of the Tropical Medicine Institute, Guangzhou University of Chinese Medicine. Informed consent was obtained at the study sites from all patients.

### HLA and KIR Genotyping

Genomic DNA was extracted from whole PBMCs and was typed for both HLA class I and KIR alleles. HLA class I genotyping was performed by a commercial sequencing service (Bioassay Laboratory of Capital Bio Corporation, Beijing, China). KIR genotyping was performed by using a multiplex PCR–sequence-specific priming (SSP) protocol, as previously described (22). Two sets of primers were designed for each of the 16 KIR genes, and the presence of the gene was detected only when both were amplified.

### Viral Load

HIV-1 RNA (copies per milliliter) measurements determined previously by a commercial kit (Real-time HIV-1, Abbott, USA) were available for analysis. To exclude the potential underestimation of viral load caused by sequence mismatch with any given primer/probe set, the undetectable viral load (<50 copies/ml) of ECs was confirmed by an ultrasensitive detection system with multiple primer/probe sets (23).

### Flow Cytometry

Commercially available monoclonal antibodies (mAbs) against the following proteins were used: CD3, CD4, CD8, CD16, CD56, KIR2DL1 (clone REA284), KIR2DL2/2DL3 (clone DX27), KIR3DL1/3DL2 (clone 5.133), KIR3DL1 (clone Dx9), KIR2DS4 (clone JJC11.6), and KIR3DS1 (clone z27).

### HIV-1 Suppression Assay

CD4^+^ T cells from each individual were purified by magnetic positive labeling (Microbeads, Miltenyi Biotec GmbH, Bergisch Gladbach, Germany) and then acutely infected with a clinical HIV-1M subtype B isolate (10^−3^ multiplicity of infection) in the presence or absence of negatively purified (Microbeads, Miltenyi Biotec) CD8^+^ T cells (>90% purity) at a CD4/CD8 ratio of 1:3, and then stimulated with SEB (0.5 μg/ml) and CD3 (5 μg/ml)/CD28 (2 μg/ml) antibodies for 16 h. After washing, the cells were cultured in quadruplicates in 96-well plates. Cultures were maintained in a final volume of 200 μl per well of RPMI 1640 medium containing 100 IU of human rIL2 (Roche Diagnostics GmbH, Mannheim, Germany) for 5 days. Viral loads were measured by a real-time RT-PCR (see above) in culture supernatants collected at day 5. The suppression assay was standardized to produce around 10^7^ (ranging from 3 × 10^6^ to 3 × 10^7^) HIV-1 RNA copies per milliliter in the 5-day culture (quadruplicates) of activated CD4^+^ T cells alone. The fold of viral suppression was determined as the geometric means of viral concentration in the culture supernatants from the infected CD4^+^ target cells only divided by the geometric means of viral concentration in the supernatants from cocultured CD8^+^ and CD4^+^ T cells. In some depletion experiments, microbead-conjugated mAbs against CD8, NK (CD16/CD56), KIR3DL1 (Dx9), and inhibitory pan-KIRs [cocktail antibodies for CD158a (KIR2DL1), CD158b (KIR2DL2/DL3), CD158f (KIR2DL5), and CD158e/k (KIR3DL1/DL2)] (Milteny Biotec) were used. Moreover, anti-KIR3DL1 (clone Dx9) and anti-KIR3DS1 (clone z27) (without Microbead conjugation) were added in the culture medium to perform neutralization experiments.

To validate the HIV-1 suppression assay, we performed the same assay with fresh PBMC samples taken from 30 healthy volunteers. The CD8^+^ T-cell-mediated viral suppression observed in all healthy volunteers was <1 log HIV-1 RNA copies per milliliter (data not shown). Therefore, the sensitivity of our viral suppression assay is determined as a reduction of 1 log HIV-1 RNA copies per milliliter.

### Cell Lysis Assay

Both purified CD8^+^ T cells (effector cells) and purified CD4^+^ T cells (target cells) pulsed with 10^10^ aldrithiol-2-treated HIV-1 M subtype B isolate (24) were labeled with 40-nM 3,3′-dihexyloxacarbocyanine (DiOC_6_) (25) (Molecular Probes) for 10 min at 37°C. Target cells were labeled with PerCP-Cy5-conjugated anti-CD4 (BD Bioscience) for 20 min on ice. After washing three times, effector cells were mixed with target cells in a U-bottomed 96-well plate at different effector/target (E/T) ratios (3:1, 1:1, and 0.3:1) in triplicate. K562 cells (target) with APC-conjugated anti-CD32 (BD Bioscience) and purified CD56^+^ (NK) cells (effector) from four HD were included as an assay control. After 4 h incubation at 37°C in the presence of SEB and anti-CD3/anti-CD28, cells were harvested and analyzed by flow cytometry. Percent cytotoxicity was calculated as follows: 100 × (% of total apoptotic target cells − % of spontaneous apoptotic target cells)/(100 − % of spontaneous apoptotic target cells).

### Statistical Analysis

Impaired data between different groups of individuals were compared by the Fisher’s Exact Test or by the Mann–Whitney.

## Results

### Most Elite Controllers Have HLA-B:Bw4-80Ile and KIR3DL1 Genotypes

Clinical and biological characteristics of the 10 ECs and the 10 HVLpts are summarized in Table S1 in Supplementary Material. All 20 untreated patients displayed a CD4^+^ T-cell count higher than 400/μl, a level that is not eligible for starting antiretroviral therapy in China.

Among the 10 ECs, 4 were homozygous for the serological motif Bw4 (No. 3, 5, 9, and 10), 5 were heterozygous for Bw4 and Bw6 (No. 1, 2, 4, 7, and 8), and the last 1 (No. 6) was homozygous for the serological motif Bw6. In the nine ECs where the Bw4 motif was present (No. 1–5 and 7–10), it displayed an isoleucine residue at position 80 (HLA-B:Bw4-80Ile) (Table 1). In comparison, among the 10 HVLpts, 7 (No. 1, 3, 5–8, and 10) were homozygous for the Bw6 motif. The remaining three (No. 2, 4, and 9) were heterozygous for the Bw6 and Bw4 serotypes among whom one (HVLpt No. 4) had the Bw4-80Ile motif and the remaining two (HVLpts No. 2 and 9) had a Bw4-80Thr motif (Table S2 in Supplementary Material).

**Table 1:** **Bw4/Bw6 motifs in 10 elite controllers (ECs) and 10 patients with high viral load (HVLpts)**.

Serological motifs	Class I type and alleles^a^	Amino-acid position (number^b^ in ECs/HVLpts)				
		77	80	81	82	83
Bw4	HLA-A/B A*2402, B*1302, B*1513, B*2702, B*2704, B*3802, B*5101, B*5102, B*5201, B*5301, B*5801	Asn (9/3)*	Ile (9/1)** Thr (0/2)	Ala (9/3)*	Leu (9/3)*	Arg (9/3)*
Bw6	HLA-B B*1501, B*1502, B*1512, B*1532, B*3505, B*4001, B*4002, B*4601, B*4801, B*5401, B*5502	Ser (5/10)*	Asn (5/10)*	Leu (5/10)*	Arg (5/10)*	Gly (5/10)*

*P < 0.05 (Fisher’s exact test was performed on the frequency comparison of single amino-acid position between ECs and HVLpts).

**P < 0.01.

*^a^Only alleles observed in the individuals who participated in the study*.

*^b^The number of individuals (either homozygous or heterozygous for Bw4 or Bw6) showing a particular amino acid at each HLA-I position*.

**Figure 1:**
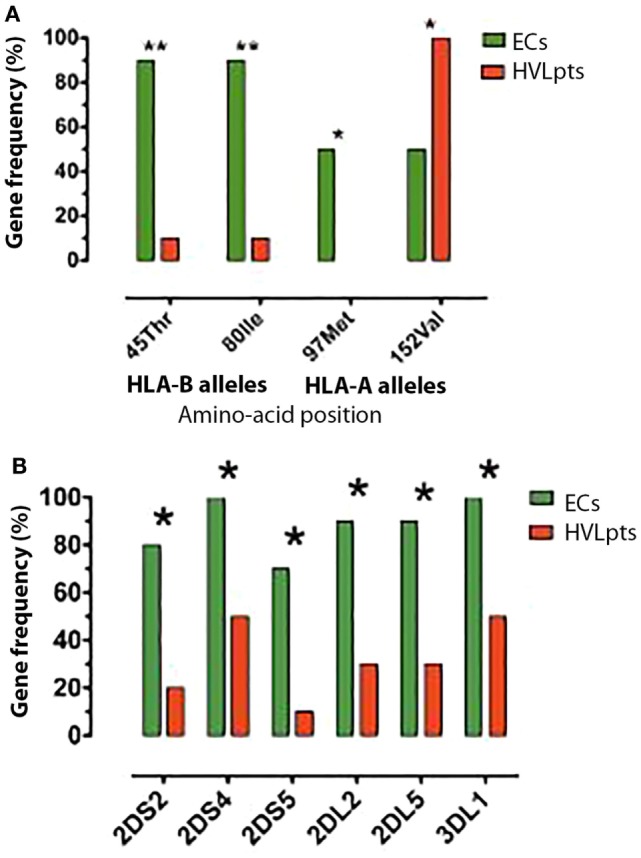
**HLA-I and KIR alleles frequencies; differences between 10 elite controllers (ECs) and 10 patients with high viral load (HVLpts)**. **(A)** Two HLA-I B and one HLA-I A alleles were more frequent in ECs (green) than in HVLpts (red); only one HLA-A allele was more frequent in HVLpts than in ECs. **(B)** Five KIR alleles were more frequent in ECs (green) than in HVLpts (red). *P < 0.05; **P < 0.01.

Overall, three HLA alleles (HLA-B 45Thr, 80Ile, and HLA-A 97Met) were significantly more frequent in ECs than in HVLpts (*P* < 0.01 for HLA-B 45Thr and 80Ile; *P* < 0.05 for HLA-A 97Met). One HLA allele (HLA-A 152Val) was significantly less frequent in ECs than in HVLpts (*P* < 0.05) (Figure 1A).

All 16 KIR genes (9 group A and 7 group B) were detected (Table S3 in Supplementary Material). The KIR group A gene frequency was equally detected in ECs (average of 8.8 genes per person) and HVLpts (8.4 genes per person) (*P* = ns), whereas the KIR group B genes were significantly more frequent in ECs (4.6 genes per person) than in HVLpts (2.2 genes per person) (*P* < 0.05).

The framework loci (KIR2DL4, KIR3DL2, and KIR3DL3) and pseudogenes (KIR2DP1 and KIR3DP1) were observed in all individuals of both groups of patients. Three inhibitory genes (KIR2DL2, KIR2DL5, and KIR3DL1) and two activating genes (KIR2DS2 and KIR2DS5) were more frequent in ECs than in HVLpts (*P* < 0.05) (Figure 1B). KIR3DL1 was carried by all 10 ECs (9 of whom having at least 1 BW4-80Ile allele) and by 5 out of the 10 HVLpts (No. 2 who was Bw6/Bw4-80Thr and No. 3, 6, 7, and 8 who were Bw6/Bw6).

### CD8^+^ T-Cells Rather than NK Cells Strongly Suppress HIV-1 Replication *In Vitro*

To exclude any possible contamination of CD8^+^ T-cells by NK cells, as NKT cells also express the CD8 molecule, CD8^+^ T-cells were purified from fresh PBMCs by deleting NK cells (CD16/CD56), monocytes (CD14), and B cells (CD19/CD20) using a mixture of microbead-conjugated mAbs.

**Figure 2:**
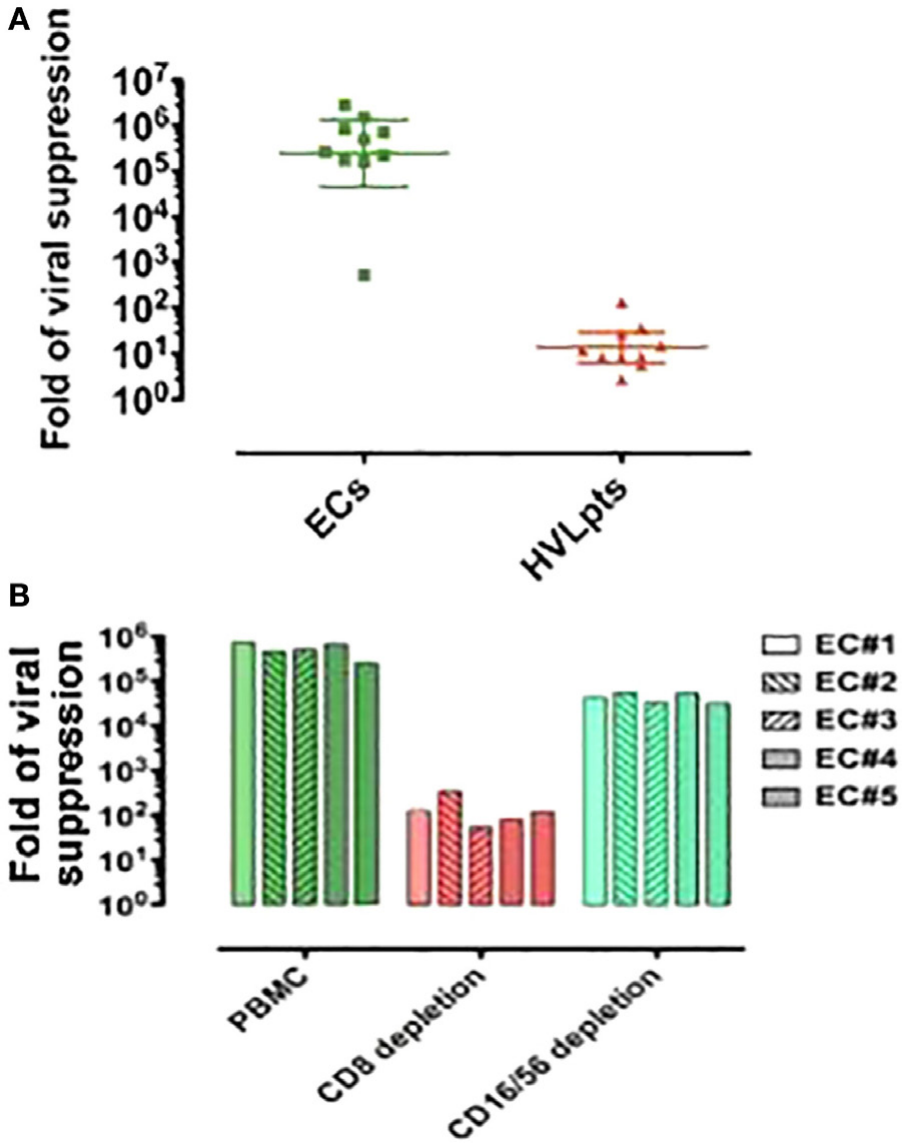
**CD8^+^ T-cell (rather than NK cell)-mediated viral suppression in 10 elite controllers (ECs) and 10 patients with high viral load (HVLpts)**. **(A)** Suppression of HIV-1 replication in autologous CD4^+^ T cells by CD8^+^ T cells purified (by negative selection) from fresh PBMCs. **(B)** By fresh PBMCs depleted of CD8 or CD16/56.

Fresh CD8^+^ T-cells from the nine ECs possessing both the HLA-Bw4-80Ile motif and the KIR3DL1 gene exhibited a strong suppression of viral replication in autologous (superinfected) target CD4^+^ T-cells (>5-log reduction of viral RNA in culture supernatants). A much weaker (3 logs) CD8^+^ T-cell-dependent viral suppression was observed for EC No. 6, who, although carrying KIR3DL1, was homozygous for HLA-B: Bw6. In contrast, the suppression of viral replication caused by fresh CD8^+^ T-cells from the HVLpts remained at baseline levels (≤2 logs) in all cases (*P* < 0.001) (Figure 2A).

Non-depleted PBMC of five ECs (two homozygous and three heterozygote for HLA-B:Bw4-80Ile but all of them carrying KIR3DL1) cocultured with autologous (superinfected) CD4^+^ T-cells suppressed strongly HIV-1 replication (>5 logs). In contrast, upon depletion of CD8^+^ T-cells, the viral suppression capacity of PBMCs dropped to nearly baseline levels (≤2 logs, *P* < 0.01). However, upon depletion of NK CD16^+^/56^+^ cells, viral suppression levels of PBMCs remained almost as high as that of non-depleted PBMC (*P* = 0.07) (Figure 2B). These data clearly indicate that CD8^+^ T-cells contribute in a much larger extent than NK cells to the inhibition of viral replication *in vitro*.

### Functions of CD8^+^ T-Cell-Mediated Viral Suppression *In Vitro*

**Figure 3:**
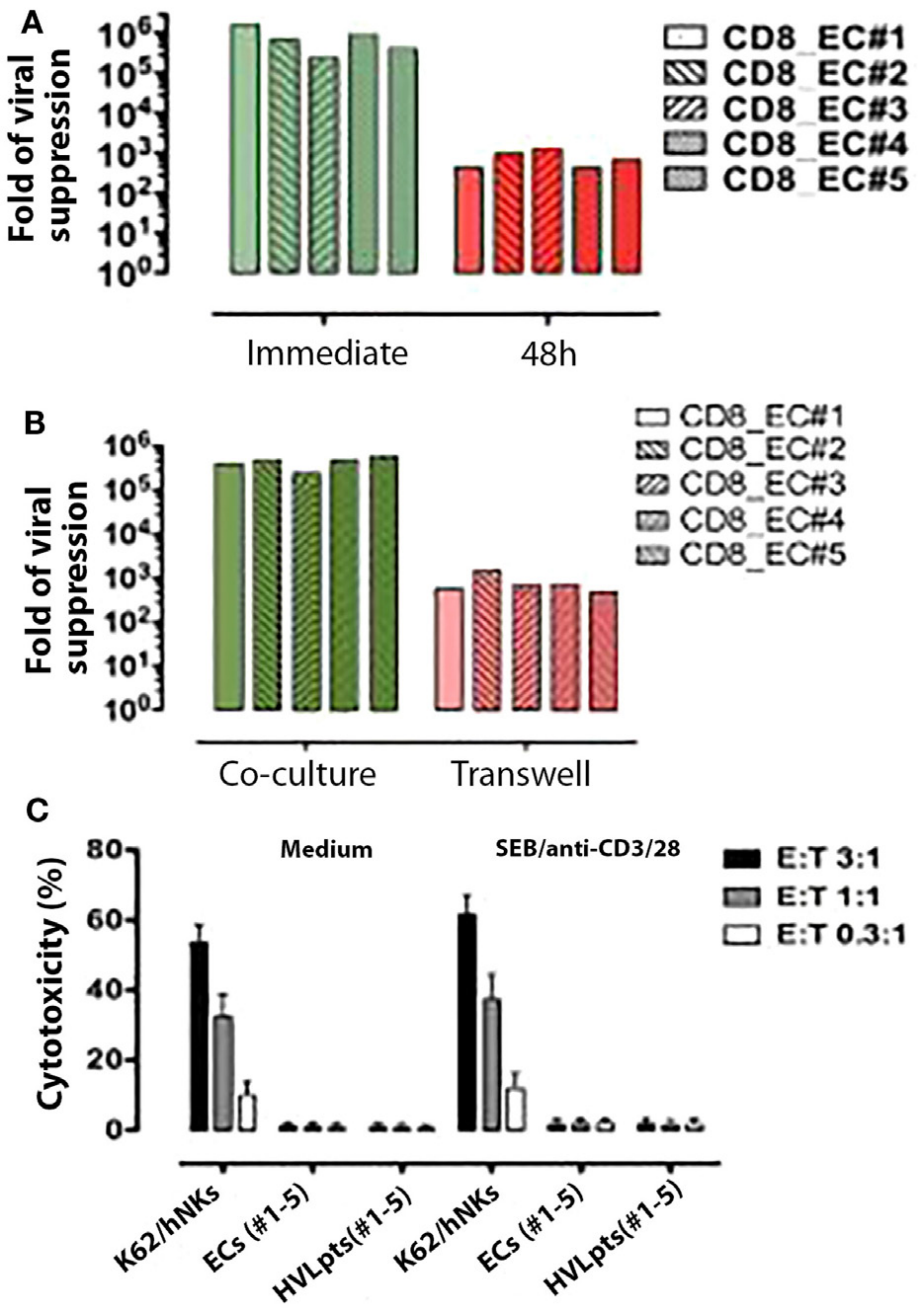
**Functional features of CD8^+^ T-cell-mediated viral suppression**. **(A)** CD8^+^ T-cell-mediated viral suppression (green) was reduced by nearly 3 logs when freshly purified CD8^+^ T cells were added 48 h after SEB and anti-CD3/28 stimulation (red). **(B)** CD8^+^ T-cell-mediated viral suppression (green) was reduced by 2.5 logs when CD8^+^ T cells were separated in a transwell culture (red). **(C)** No cytotoxicity was observed in autologous HIV-1-infected CD4^+^ T cells (target cells) co-incubated with CD8^+^ T-cells (effector cells) from ECs No. 1–5.

When freshly purified CD8^+^ T-cells (stimulated at the same time as target CD4^+^ T-cells with SEB and anti-CD3/anti-CD28 antibodies) were added 48 h after the activation of HIV-1-superinfected CD4^+^ T-cells, viral suppression was much less effective (<3 logs) compared to that obtained when CD8^+^ T-cells were added before T-cell activation where viral suppression was maximum (>5 logs) (*P* < 0.05) (Figure 3A). This observation indicates that the inhibition of viral replication by CD8^+^ T-cells is more efficient when CD4^+^ T-cells are quiescent/not activated.

To investigate whether the viral suppressive effect of CD8^+^ T-cells would involve soluble factors or rather require cell-to-cell contact, experiments were conducted in transwell culture chambers, in which target CD4^+^ T-cells were physically separated from effector CD8^+^ T-cells. Under these experimental conditions, the suppression of HIV-1 replication was strongly impaired (reaching only <3 logs), supporting the hypothesis that CD8^+^ T-cell-mediated viral suppression requires cell-to-cell contact (Figure 3B).

We next investigated whether CD8^+^ T-cell-mediated viral suppression would involve anti-CD4^+^ cytotoxic effects. A highly sensitive cytotoxicity assay (25) was used to detect cell lysis in coculture experiments mixing autologous CD8^+^ T-cells and HIV-1-superinfected target CD4^+^ T-cells derived from five ECs (No. 1–5) and five HVLpts (No. 1–5). No lysis could be documented under these conditions (Figure 3C), indicating that the CD8-mediated viral suppression observed in ECs is most likely not cytolytic.

### HLA and KIR Interaction in CD8^+^ T-Cell-Mediated Viral Suppression

The molecular basis of viral suppression by autologous or allogeneic CD8^+^ T-cells carrying KIR3DL1 (but not KIR3DS1) genes was investigated in more details in four ECs with different HLA-B genotypes: ECs No. 1 and 2 were heterozygote Bw4-80Ile/Bw6, EC No. 3 was homozygote Bw4-80Ile/Bw4-80Ile, while EC No. 6 had no Bw4-80Ile allele (since this patient was homozygote Bw6/Bw6). Superinfected CD4^+^ T-cells taken from these four ECs and from four HVLpts [No. 1 and 3: both homozygotes Bw6/Bw6, No. 2: heterozygote Bw6/Bw4-80Thr, and No. 4: Bw6/Bw4-80Ile (without a KIR3DL1 allele)] were used as targets. Two HDs carrying KIR3DL1 (but without KIR3DS1) and HLA-B Bw4-80Ile genes were included as control.

**Figure 4:**
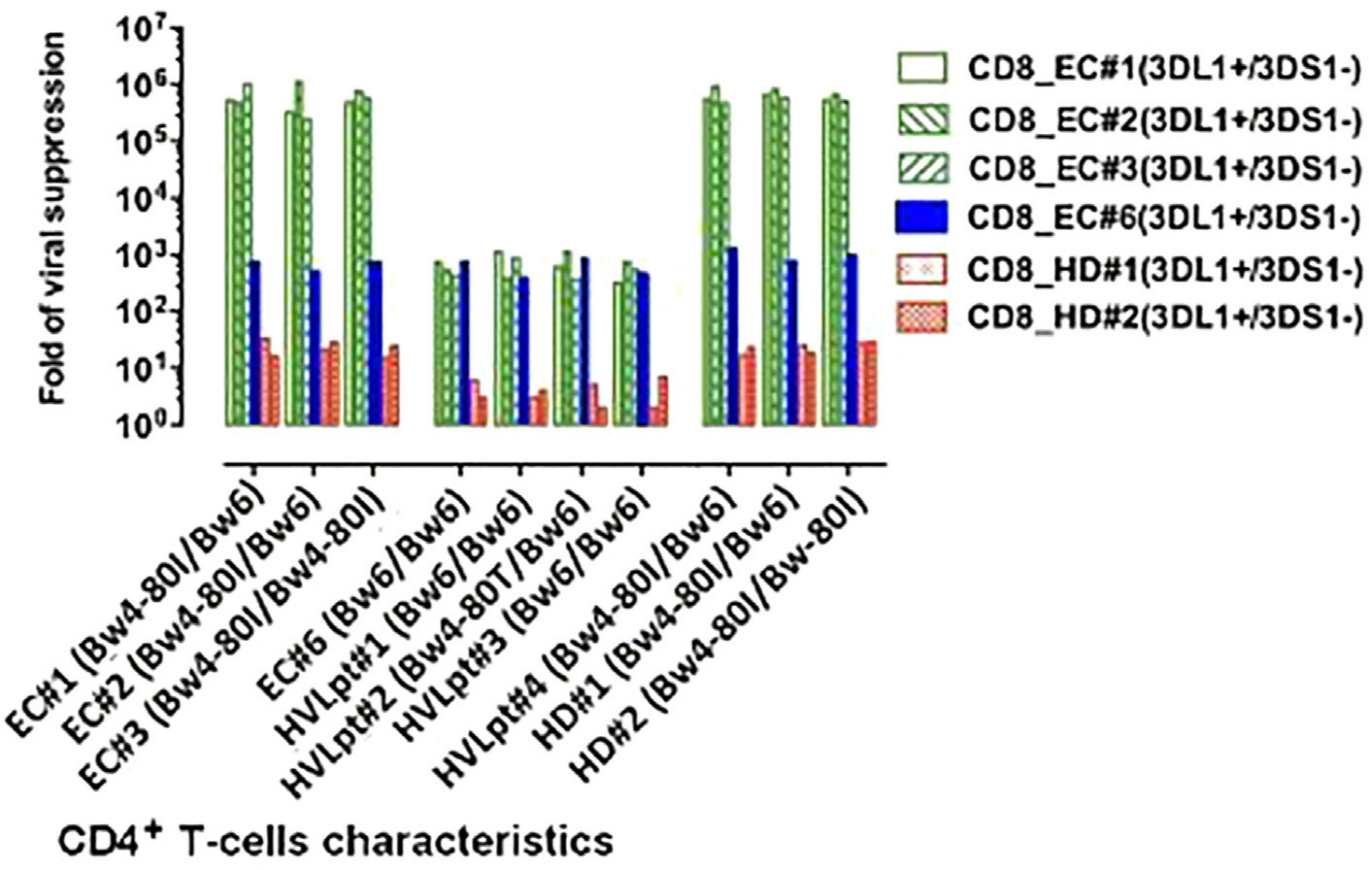
**Association between CD8^+^ T-cell-mediated viral suppression and HLA-B Bw4-80Ile/KIRsDL1 combined genotypes**. For details, see Section “HLA and KIR Interaction in CD8^+^ T-Cell-Mediated Viral Suppression” in Section “Results.”

In ECs No. 1–3 (who carried at least one Bw4-80Ile allele and were KIR3DL1), the CD8^+^ T-cell-mediated viral suppression was very strong (5–6 logs) whether target CD4^+^ T-cells were autologous or allogeneic (Figure 4, left green bars), In contrast, CD8^+^ T-cells from the same EC No. 1–3 had a much lower suppressive activity (barely reaching 3 logs) on target CD4^+^ T-cells of EC No. 6 or on those of HVLpts No. 1–3 who did not carry Bw4-80Ile (since they were Bw6/Bw6 or Bw6/Bw4-80Thr) (Figure 4, middle, green bars). Moreover, as already shown (Figure 2A), the same low level of viral suppression was achieved when EC No. 6 CD8^+^ T-cells were cocultured with their autologous target CD4^+^ T cells; EC No. 6 carried the KIR3DL1 gene but had no Bw4-80Ile allele (he was Bw6/Bw6). Interestingly, although EC No. 6 did carry the KIR3DL1 gene, his CD8^+^ T-cells also induced a low level of viral suppression when cocultured with HLA-B:Bw4-80Ile-carrying target CD4^+^ T cells from ECs No. 1–3 (Figure 4, blue bar); a baseline (1 log) viral suppression was finally observed in HLA-B:Bw4-80Ile-carrying target CD4^+^ T cells cocultured with KIR3DL1-carrying CD8^+^ T-cells from HDs (Figure 4, red bars). On the other hand, a strong CD8^+^ T-cell-mediated viral suppression was observed with allogeneic target CD4^+^ T-cells of HVLpt No. 4 and of HDs No. 1 and 2, because all three individuals carried at least one Bw4-80Ile allele, while the effector CD8^+^ T-cells carried the KIR3DL1 gene (Figure 4, right, green bars).

### KIR3DL1-Expressing CD8^+^ T-Cells Responsible for HIV-1 Suppression

**Figure 5:**
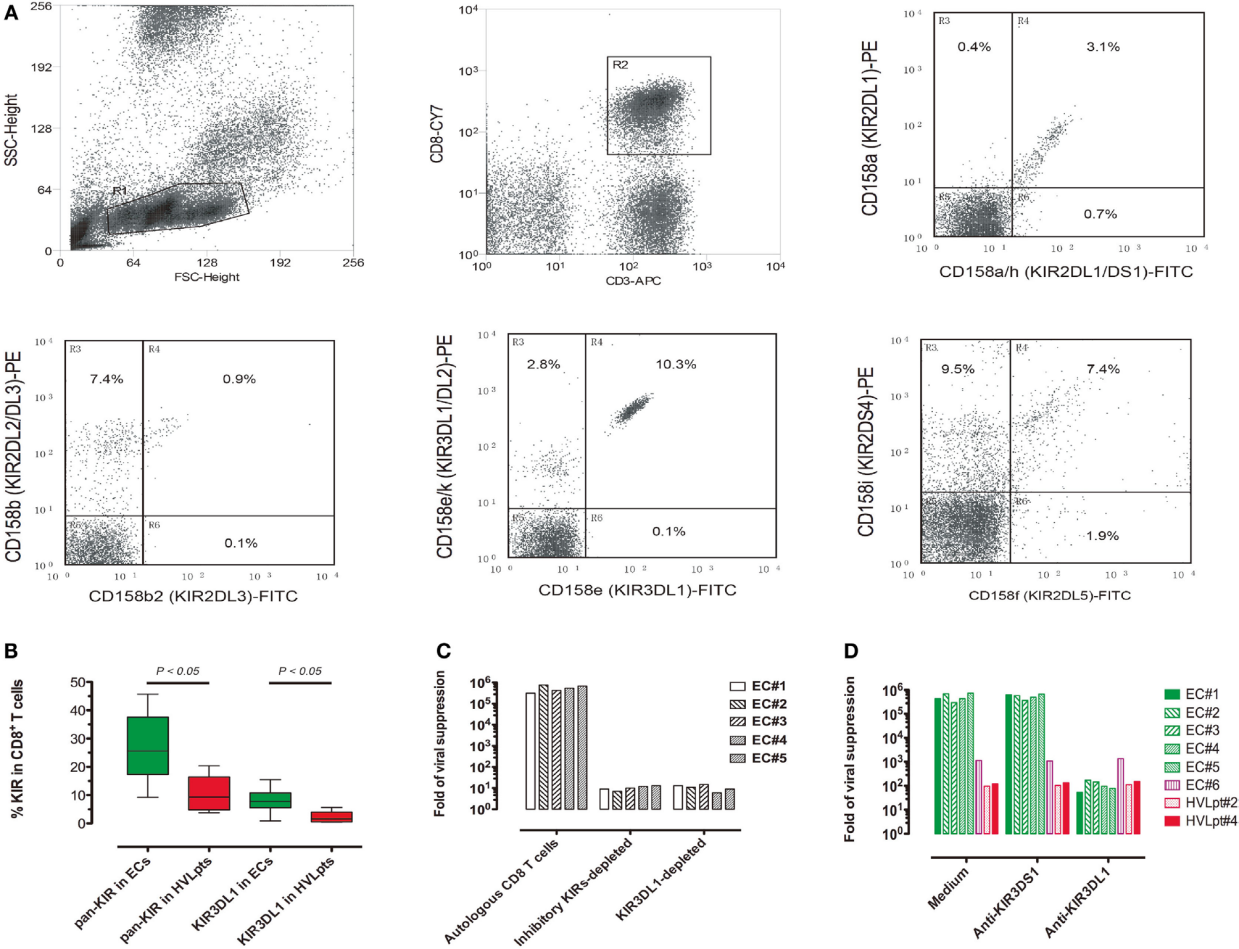
**KIR3DL1 expression on CD8^+^ T-cells is required for HLA-B:Bw4-80Ile-dependent viral suppression**. **(A)** Cytometric analysis of KIR expression in CD8^+^ T-cells freshly taken from a representative EC (No. 3). **(B)** Pan-KIR (total inhibitory KIRs) and KIR3DL1-expressing CD8^+^ T-cells were significantly higher in ECs (No. 1–5) (green) than in HVLpts carrying KIR3DL1 gene (No. 2, 3, and 6–8). **(C)** CD8^+^ T-cell-mediated viral suppression was reduced by 4–5 logs when freshly pan-KIR or KIR3DL1-depleted CD8^+^ T-cells were added as compared with bulk CD8^+^ T-cells. **(D)** CD8^+^ T-cell-mediated viral suppression was reduced by 3–4 logs in Bw4-80I/KIR3DL1-matched patients (EC#1–5) when the anti-KIR3DL1 antibody (clone Dx9) was added as compared with parallel cultures in the presence of anti-KIR3DS1 antibody (clone z27) or medium alone. In contrast, the low levels of viral suppression observed in Bw4-80I/KIR3DL1-mismatched patients (EC#6, HVLpt#2, and HVLpt#4) remained unaffected by the anti-KIR3DL1 antibody (clone Dx9).

Having observed the correlation between the combination of KIR3DL1 and HLA-B:Bw4-80Ile genes and HIV-1 suppression *in vitro* and *in vivo*, we further investigated the expression of KIRs on CD8^+^ T-cells in the 10 ECs (No. 1–10) and the 5 HVLpts carrying KIR3DL1 (No. 2, 3, and 6–8). On average, the mean expression of all inhibitory KIRs (including KIR2DL1, KIR2DL2, KIR2DL3, KIR2DL5, KIR3DL1, and KIR3DL2, hereafter collectively referred as pan-KIR) in the CD8^+^ T-cells from the 10 ECs was 27.1% (range 9.2–45.7%). The expression of pan-KIR in the CD8^+^ T-cells from the 5 HVLpts (mean 10.3%, range 3.8–20.4%) was significantly lower (*P* < 0.05). The same was true for the expression of KIR3DL1 in the CD8^+^ T-cells from the 10 ECs (mean 8%, range 0.9–15.5%), a percentage significantly higher than that observed in HVLpts (mean 2.2%, range 0.5–5.7%, *P* < 0.05) (Figures 5A,B; Table S4 in Supplementary Material).

Due to the fact that only 8% (range 0.9–15.5%) of ECs’ CD8^+^ T-cells expressed KIR3DL1 at the cell membrane surface, it is very difficult to purify KIR3DL1-expressing CD8^+^ T-cells from such a small population (<3%) of PBMCs (which would have allowed to perform specific functional assays). Alternatively, we conducted viral suppression assays using fresh CD8^+^ T-cells from ECs (No. 1–5) depleted (or not) of KIR-expressing cells by microbead-conjugated mAbs against KIRs. CD8^+^ T-cell-mediated suppression of HIV-1 replication in Bw4-80Ile-carrying CD4^+^ T-cells disappeared when effector CD8^+^ T-cells were depleted of either whole inhibitory KIRs or KIR3DL1 alone (Figure 5C).

In addition, we performed the viral suppression assay in the presence (or absence) of anti-KIR3DL1 or anti-KIR3DS1 antibody to check whether the viral suppression was actually influenced by this receptor. CD8^+^ T-cell-mediated suppression of HIV-1 replication was suppressed by the anti-KIR3DL1 antibody (and not in the presence of an anti-KIR3DS1 antibody) in the five Bw4-80I/KIR3DL1-matched ECs (ECs#1–5) (Figure 5D, green bars); in contrast, the low levels of viral suppression observed in the three Bw4-80I/KIRD3L1-mismatched patients (EC#6, Bw4-80I^−^/KIR3DL1^+^; HVLpt#2, Bw4-80I^−^/KIR3DL1^−^; and HVLpt#4, Bw4-80I^+^/KIR3DL1^−^) were not modified by the presence of the anti-KIR3DL1 antibody (Figure 5D, red bars). These observations confirm the specificity of the anti-KIR3DL1 antibody to block the suppression of HIV-1 replication observed in Bw4-80I/KIR3DL1-matched ECs. These data suggest that KIR3DL1-expressing CD8^+^ T-cells are effectively responsible for switching off the activation of HIV-1-infected HLA-B:Bw4-80Ile-carrying CD4^+^ T-cells, thereby suppressing viral replication in superinfected target cells.

## Discussion

Our data demonstrate that fresh inhibitory KIR3DL1-expressing CD8^+^ T cells from HIV-1-infected ECs inhibit virus replication in autologous or allogeneic HIV-1-infected Bw4-80Ile^+^ CD4^+^ T-cells. Although our observations cannot proof the existence of a single protein interaction between Bw4-80Ile and KIR3DL1, the deletion of KIR3DL1-expressing cells and the neutralization of KIR3DL1 by an anti-KIR3DL1 mAb strongly suggest the major role of the Bw4-80I/KIR3DL1 interaction in the control of viral replication by ECs’ CD8^+^ T-cells. This specific cooperation between effector and target cells seems to prevent CD4^+^ T-cell activation and results ultimately in the strong suppression of HIV-1 replication. Interestingly, in the context of the *in vitro* assay used in the present study, the cytotoxic role of CD8^+^ T-cells (26) is nil and that of suppressive soluble factors (27) appears likely marginal (Figures 3 and 4). Overall, these findings provide the first evidence for a pivotal role of Bw4-80Ile-restricted KIR3DL1-expressing CD8^+^ T-cells in the natural control of HIV-1 replication in ECs, highlighting for the first time a mechanistic basis for the protective effect of combined Bw4-80Ile and KIR3DL1 genotypes, which was reported in several studies of molecular epidemiology (2–4).

In healthy individuals, 5% (range 1–38%) of CD8^+^ T-cells express all inhibitory KIRs (pan-KIR) (12). In the present study, we observed that as high as 27.1% (range 9.2–45.7%) of CD8^+^ T-cells expressed the pan-KIR in ECs as compared to 10.3% (range 3.8–20.4%) in HVLpts (Figure 5B; *P* < 0.01). Of note is the fact that we detected the pan-KIR with mAbs specific for six inhibitory KIRs (KIR2DL1, KIR2DL2, KIR2DL3, KIR2DL5, KIR3DL1, and KIR3DL2), whereas the pan-KIR expression was previously analyzed using mAbs specific for only four inhibitory KIRs (KIR2DL1, KIR2DL3, KIR3DL1, and KIR3DL2); the levels of pan-KIR staining should thus be higher in our study than in the previous report. The higher level of pan-KIR expression in ECs than in HVLpts could be explained partially by the observation that both KIR2DL2 and KIR2DL5 (exclusively included in our pan-KIR panel) genes were more frequent in ECs than in HVLpts (Figure 1B). However, although equally carrying KIR3DL1 gene, the expression of KIR3DL1 in CD8^+^ T-cells was significantly higher in ECs (8.9%) than in HVLpts (2.2%) (*P* < 0.05). This finding indicates that an expansion of KIR3DL1-expressing CD8^+^ T-cells triggered by HIV-1-infected HLA-B:Bw4-80Ile^+^CD4^+^ T-cells may occur during the natural course of HIV-1 infection in ECs.

NK cells have been shown to play a critical role in the initial containment of viral replication in several viral infections (28). The observation of their expansion during the acute phase of HIV-1 infection has suggested a potential role for the innate immune response in the initial control of HIV-1 replication (29). In this context, NK cells have been reported to inhibit HIV-1 replication in an HLA-B Bw4-80Ile/KIR 3DS1- or KIR 3DL1-dependent manner (16, 17). However, in ECs, the suppression of replication induced by CD8^+^ T-cells was shown to be more robust than that mediated by NK cells (18). Our study fully confirmed this observation: the viral suppression levels of PBMCs predepleted of NK cells (CD16/CD56) was reduced only by 1 log as compared to bulk PBMCs (*P* = 0.07) in the same culture system (Figure 2B).

From the observations reported here, we can conclude that the long-term containment of HIV-1 in ECs mostly relies on the combined presence in these patients of HIV-1-infected target CD4^+^ T-cells expressing Bw4-80Ile and of effector CD8^+^ T-cells expressing KIR3DL1. This finding is consistent with the demonstration that KIR-expressing CD8^+^ T-cells are terminally differentiated effectors with a restricted KIR repertoire dominated by a single KIR, the specificity of which is often distinct from that of the same patient’s NK cells (12). In addition, previous studies have also indicated that the engagement of inhibitory KIRs on CD8^+^ T-cells drives the accumulation of KIR^+^ memory CD8^+^ T-cells by downregulating activation-induced cell death (30, 31). Thus long-lived memory KIR-expressing CD8^+^ T-cells might exist as demonstrated for NKT cells (32). The moderate suppression of viral replication (almost 3 logs) observed in the HIV-1 (CRF01_AE subtype)-infected EC No. 6 (Figure 2A), who was homozygous for the Bw6 serologic motif (HLA-B*4601/4801) (Table S2 in Supplementary Material), does not contradict the above conclusions, since some Bw6 alleles (such as HLA-B*14, HLA-B*4201, and HLA-B*8101) were also reported to be associated with delayed disease progression in individuals with HIV-1 B and C subtypes (33). Here, the protection might involve other mechanisms, such as the recognition of immunodominant HIV-1 peptides in the classical acquired immune response setting (34, 35). Nevertheless, due to the limited number of available samples analyzed in the present study, definitive conclusions need to be confirmed in future studies with larger sample sizes.

The low suppression of viral replication (3 logs) observed both in transwell experiments (Figure 3B) and in mismatched cocultures of patients’ CD4 and CD8^+^ T-cells (Figure 4, green bars of the middle panel and blue bars) remains to be explained. Such a low viral suppression stands, however, nearly 2 logs above the baseline suppression (1 log), observed in coculure of uninfected donors’ CD8^+^ T-cells together with patients’ CD4^+^ T-cells. Whether soluble factors may have a contribution in the overall viral suppression induced by KIR3DL1-expressing CD8^+^ T-cells remains to be explored. This phenomenon would however not be surprising since effector KIR3DL1-expressing CD8^+^ T-cells have been suggested to execute their suppressive activity through secretions of cytokines or chemokines as demonstrated for NK cells (28).

Although the stimulation of CD4^+^ T-cells with SEB and anti-CD3/CD28 antibodies in our viral suppression assay may cause activation of CD8^+^ T-cells and thus improve their suppressive function (36), this cannot explain the results showing that the suppression was stronger when CD8^+^ T-cells were added before than after CD4^+^ T-cells stimulation with SEB and anti-CD3/CD28 antibodies since CD8^+^ T-cells were always stimulated in parallel at the same time as CD4^+^ T-cells whether added before or after the activation of CD4^+^ T-cells. This observation suggests that the inhibition of viral replication by KIR3DL1-expressing CD8^+^ T-cells is much more efficient when target CD4^+^ T-cells carrying Bw4-80I gene are quiescent/not activated. In a previous study using autologous-infected CD4^+^ T-cells elimination as a measure of the suppressive activity of CD8^+^ T-cells, it was shown that activated CD8^+^ T-cells from ECs were capable to kill infected (P24^+^) CD4^+^ T-cells (36). This result is in contradiction with our observation that cell lysis was not detected in coculture experiments mixing autologous CD8^+^ T-cells and HIV-1-superinfected target CD4^+^ T-cells derived from five ECs (No. 1–5) and five HVLpts (No. 1–5) (Figure 3C). This contradiction might be explained by the use of a laboratory-adapted HIV-1 strain (HIV_SF162_) with a high infection rate (>30% of CD4^+^ T-cells) in the abovementioned study (36), while it was a clinical HIV-1 isolate with a much lower infection rate (<5%), which was used in the present study.

So far, immunological studies in HIV-1 infection have mainly focused on classical virus-specific cytotoxic T cells (CTLs) and antibody responses (26). Consequently, the goal of most HIV vaccines developed during the last two decades was to induce long-lived memory B and T cells capable of protecting the host from infection *via* the production of high-affinity antibodies and/or CTLs (37). However, efforts aimed at stimulating such approaches to develop a vaccine against HIV-1 have been so far unsuccessful, possibly because most vaccine prototypes were aimed at rapidly activating CD4^+^ T-cells after HIV-1 infection. However, because CD4^+^ T-cells are themselves the privileged target of HIV-1, their fast activation in the presence of the virus might instead facilitate HIV replication (38).

Interestingly, the present findings provide a mechanistic background for our recent observation in SIV-infected Chinese macaques (39, 40). In these studies, we have reported that regulatory/suppressive CD8^+^ T-cells induced by an oral vaccine could suppress the activation of SIV-positive CD4^+^ T-cells, prevent viral replication in these cells, and protect the animals against subsequent SIV challenge. In the present study, we demonstrated that a similar population of regulatory/suppressive CD8^+^ T-cells naturally exists, that it can inhibit the activation of HIV-1-infected cells and allow the persistent suppression of HIV-1 replication in human ECs. A difference with the animal model, however, is the fact that suppressive CD8^+^ T-cells developed by vaccinated Chinese macaques were MHC-1B-E restricted, while the role of HLA-E restriction seems less clear in human ECs (Figure S1 in Supplementary Material). Whether such a discrepancy results from a distinct epitope associated with the mAbs we used remains to be determined. Of note in this context that the regulatory/suppressive CD8^+^ T-cells (and their resulting *in vivo* protection) observed in vaccinated macaques of Chinese origin have neither been found in macaques of North China origin (data not shown) nor in those of Indian origin (G. Silvestri, Cent Gardes conference: HIV vaccines, Annecy, France, October 25–27, 2015) similarly immunized.

In conclusion, we have reported that in most ECs, the principal mechanisms of suppression of HIV-1 replication depend on specific genetic features regulating the interaction of effector CD8^+^ T-cells with target-infected CD4^+^ T-cells. Taken together with the observation that regulatory/suppressive CD8^+^ T-cells are generated in vaccinated Chinese macaques (39, 40), these data provide a major input for the design of an effective HIV-1 vaccine in humans.

## Author Contributions

WL and J-MA were responsible for the overall study design, organization, data analyses, and writing of the paper. SC, CL, JK, HF, HD, ML, JF, and WG, assisted by LF, participated in the study design and performed experiments.

## Conflict of Interest Statement

J-MA and WL have received grants from and are shareholders of Biovaxim Ltd. The other co-authors report no conflicts of interest.
