# Supplementary material for: Suppression of HIV Replication by CD8+ Regulatory T-Cells in Elite Controllers
Source: Front Immunol. 2016 Apr 18;7:134. doi: 10.3389/fimmu.2016.00134 (PMC4834299; doi:10.3389/fimmu.2016.00134)
Supplement: Supplementary file 1 [file table_1.docx]

**Table S1.** Clinical and Biological Characteristics of 10 elite controllers (ECs) and 10 patients with high viral load (HVLpts) recruited from a cohort of untreated HIV-1-seropositive patients at the CDC of Xishuangbanna,Yunnan ( China).

Patient Sex Transmission HIV-1M CD4^+^ T-cell Plasma viral load

ID routes subtypes count (µl^-1^) (RNA copies/ml)

EC-#1 M Heterosexual CRF01_AE 871 <50

EC-#2 F Heterosexual CRF01_AE 696 <50

EC-#3 F Heterosexual CRF01_AE 517 <50

EC-#4 F Heterosexual CRF01_AE 753 <50

EC-#5 M Heterosexual CRF01_AE 628 <50

EC-#6 M IDU CRF01_AE 614 <50

EC-#7 F Heterosexual CRF01_AE 583 <50

EC-#8 F Heterosexual CRF01_AE 921 <50

EC-#9 F Heterosexual CRF01_AE 779 <50

EC-#10 F Heterosexual CRF01_AE 635 <50

HVL-#1 F Heterosexual CRF01_AE 591 22.600

HVL-#2 F Heterosexual CRF01_AE 433 33.100

HVL-#3 M Heterosexual CRF01_AE 442 102.100

HVL-#4 F Heterosexual CRF01_AE 438 15.600

HVL-#5 F Heterosexual CRF01_AE 466 16.500

HVL-#6 F Heterosexual CRF01_AE 414 21.300

HVL-#7 F Heterosexual CRF01_AE 499 52.000

HVL-#8 M IDU CRF01_AE 513 30.800

HVL-#9 F Heterosexual CRF01_AE 492 19.900

HVL-#10 M Heterosexual CRF01_AE 498 28.400
