# Supplementary material for: Suppression of HIV Replication by CD8+ Regulatory T-Cells in Elite Controllers
Source: Front Immunol. 2016 Apr 18;7:134. doi: 10.3389/fimmu.2016.00134 (PMC4834299; doi:10.3389/fimmu.2016.00134)
Supplement: Supplementary file 2 [file table_2.docx]

**Table S2.** HLA-I alleles of 10 elite controllers (ECs) and 10 patients with high viral load (HVLpts).

Patient ID HLA-A HLA-B (serotypes) HLA-C

EC-#1 A*0207/3303 B*4601/5801 (Bw6/Bw4) C*0102/0302

EC-#2 A*1101//1101 B*4601/5201 (Bw6/Bw4) C*0102/0702

EC-#3 A*2402/3303 B*5201/5301 (Bw4/Bw4) C*0702/1202

EC-#4 A*0301/1101 B*1502/5201 (Bw6/Bw4) C*0801/1202

EC-#5 A*1101/7402 B*2702/5102 (Bw4/Bw4) C*1202/1402

EC-#6 A*0207/1101 B*4601/4801 (Bw6/Bw6) C*0102/0801

EC-#7 A*2402/2402 B*1502/5102 (Bw6/Bw4) C*0801/1502

EC-#8 A*3001/3303 B*1302/5801 (Bw4/Bw4) C*0302/0602

EC-#9 A*1101/1101 B*5201/5301 (Bw4/Bw4) C*0304/1203

EC-#10 A*0203/2402 B*1513/5201 (Bw4/Bw4) C*0702/0801

HVLpt-#1 A*0207/0207 B*4601/4601 (Bw6/Bw6) C*0102/0102

HVLpt-#2 A*1101/1101 B*1532/3802 (Bw6/Bw4) C*0304/0702

HVLpt-#3 A*1101/1102 B*4001/5502 (Bw6/Bw6) C*0702/1203

HVLpt-#4 A*0207/1103 B*4601/5101 (Bw6/Bw4) C*0102/0702

HVLpt-#5 A*0207/2407 B*1501/3505 (Bw6/Bw6) C*0303/0401

HVLpt-#6 A*1101/1101 B*1501/4001 (Bw6/Bw6) C*0304/0702

HVLpt-#7 A*0207/1101 B*1502/4601 (Bw6/Bw6) C*0102/0801

HVLpt-#8 A*1101/1101 B*4001/5401 (Bw6/Bw6) C*0702/1203

HVLpt-#9 A*0203/2402 B*1512/2704 (Bw6/Bw4) C*0303/1202

HVLpt-#10 A*0207/1101 B*4002/4801 (Bw6/Bw6 C*0702/0822
