# Supplementary material for: Suppression of HIV Replication by CD8+ Regulatory T-Cells in Elite Controllers
Source: Front Immunol. 2016 Apr 18;7:134. doi: 10.3389/fimmu.2016.00134 (PMC4834299; doi:10.3389/fimmu.2016.00134)
Supplement: Supplementary file 3 [file table_3.docx]

**Table S3**. Killer immunoglobulin-like receptor (KIR) genotypes of 10 elite controllers (ECs) and 10 patients with high viral load (HVLpts).

Gene names Groups ECs HVLpts *P* value

2DL1 A 10 10 1.0000

2DL2 B 9 3 0.0198*

2DL3 A 10 10 1.0000

2DL4 A 10 10 1.0000

2DL5 B 9 3 0.0198*

2DS1 B 6 5 1.0000

2DS2 B 8 2 0.0230*

2DS3 B 2 3 1.0000

2DS4 A 10 5 0.0325*

2DS5 B 7 1 0.0198*

3DL1 A 10 5 0.0325*

3DL2 A 10 10 1.0000

3DL3 A 10 10 1.0000

3DS1 B 5 5 1.0000

2DP1 A 10 10 1.0000

3DP1 A 10 10 1.0000

**P* < 0.05 (Fisher’s Exact Test).
