# Supplementary material for: Suppression of HIV Replication by CD8+ Regulatory T-Cells in Elite Controllers
Source: Front Immunol. 2016 Apr 18;7:134. doi: 10.3389/fimmu.2016.00134 (PMC4834299; doi:10.3389/fimmu.2016.00134)
Supplement: Supplementary file 4 [file table_4.docx]

**Table S4.** The Expression of KIRs in the CD3^+^CD8^+^ cells freshly taken from the 10 elite controllers (ECs) and the five patients with high viral load (HVLpts) with the KIR3DL1 gene.

**Patient ID % of pan-KIR^+^ cells % of KIR3DL1^+^ cells**

EC group (n = 10)

EC#1 22.8 8.2

EC#2 34.4 9.8

EC#3 25.9 10.3

EC#4 38.3 15.5

EC#5 45.7 6.7

EC#6 18.7 0.9

EC#7 9.2 6.8

EC#8 25.4 12.4

EC#9 37.3 7.4

EC#10 13.1 2.2

**Mean ± SD 27.08 ± 11.73 8.02 ± 4.37**

HVLpt group (n = 5)

HVLpt#2 9.3 0.7

HVLpt#3 12.5 5.7

HVLpt#6 20.4 0.5

HVLpt#7 5.7 1.6

HVLpt#8 3.8 2.3

**Mean ± SD 10.34 ± 6.54 2.16 ± 2.11**

***P* value (ECs vs HVLpts) 0.0127 0.0127**
