# Supplementary material for: Suppression of HIV Replication by CD8+ Regulatory T-Cells in Elite Controllers
Source: Front Immunol. 2016 Apr 18;7:134. doi: 10.3389/fimmu.2016.00134 (PMC4834299; doi:10.3389/fimmu.2016.00134)

Figure S1. Relatedness between CD8$^+$T-cell-mediated viral suppression and classical HLA-I ABC and non classical HLA-I E

Viral suppression in autologous target CD4$^+$T-cells by CD8$^+$T-cells freshly purified from PBMCs of elite controllers N°1 to 5 (green) was reduced by 1 log in the presence of monoclonal antibody against HLA-I E antigen ($P < 0.01$) (red) but remained unchanged in the presence of monoclonal antibodies against HLA-I ABC (pale green).

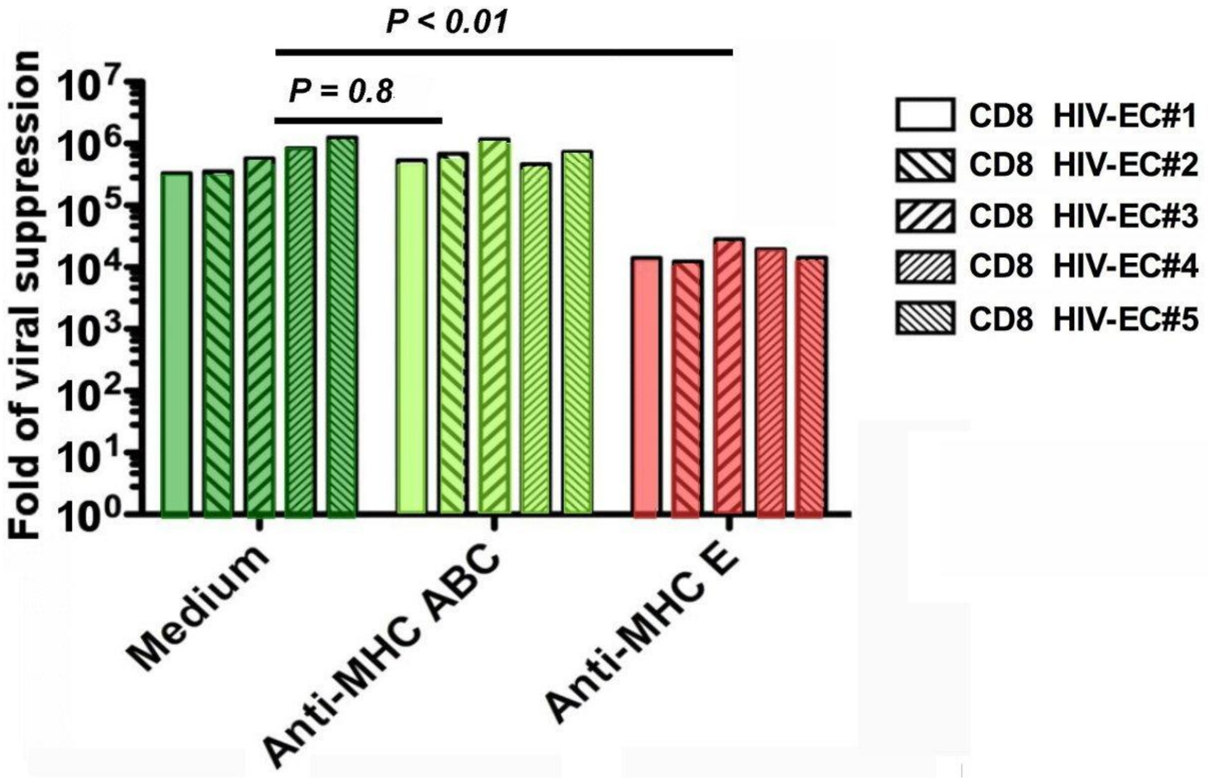

Supplement: Supplementary file 5 [file image_1.PDF]
